# Increased ATG5 Expression Predicts Poor Prognosis and Promotes EMT in Cervical Carcinoma

**DOI:** 10.3389/fcell.2021.757184

**Published:** 2021-11-25

**Authors:** Suna Zhou, Xuequan Wang, Jiapei Ding, Haihua Yang, Youyou Xie

**Affiliations:** ^1^ Laboratory of Cellular and Molecular Radiation Oncology, The Affiliated Taizhou Hospital, Wenzhou Medical University, Taizhou, China; ^2^ Department of Radiation Oncology, The Affiliated Taizhou Hospital, Wenzhou Medical University, Taizhou, China

**Keywords:** autophagy-related genes, Atg5, autophagy, EMT, cervical cancer, prognosis

## Abstract

Cervical cancer has the second-highest incidence and mortality of female malignancy. The major causes of mortality in patients with cervical cancer are invasion and metastasis. The epithelial–mesenchymal transition (EMT) process plays a major role in the acquisition of metastatic potential and motility. Autophagy-related genes (ARGs) are implicated in the EMT process, and autophagy exerts a dual function in EMT management at different phases of tumor progression. However, the role of specific ARGs during the EMT process has not yet been reported in cervical cancer. Based on the data from the *Cancer* Genome Atlas (TCGA) cervical squamous cell carcinoma and endocervical adenocarcinoma (CESC) sequencing database, we performed the prognosis analysis for those ARGs obtained from the Human Autophagy database. ATG5 was identified as the only important harmful marker influencing survival of cervical cancer patients by univariate Cox regression (HR 1.7; 95% CI: 1.0–2.8, *p* = 0.047), and the 5-years survival rate for the high- and low-ATG5 expression groups was 0.486 (0.375–0.631) and 0.782 (0.708–0.863), respectively. TCGA CESC methylation data showed that eight methylation sites of ATG5 could also be significantly associated with the overall survival (OS) of cervical cancer patients. Single-sample gene-set enrichment and gene functional enrichment results showed that ATG5 was correlated with some cancer-related pathways, such as phagocytosis-related genes, endocytosis-related genes, immune-related genes, EMT score, and some EMT signature-related genes. Next, cell migration and invasion assay and Western blot were applied to detect the function of ATG5 in EMT of cervical cancer. In cervical cancer cells, ATG5 knockdown resulted in attenuation of migration and invasion. The functional study showed that knockdown of ATG5 could reverse EMT process by P-ERK, P-NFκBp65, P-mTOR pathways, and so on. In conclusion, the present study implies that ATG5 was a major contributor to EMT regulation and poor prognosis in cervical cancer.

## Introduction

Cervical cancer secondary to breast cancer has the second-highest incidence and mortality of cancer in the female with over 604,127 new cases and 341,831 deaths worldwide per year (Sung et al., 2021). Cervical squamous cell carcinoma and endocervical adenocarcinoma (CESC) are the main pathological types of cervical cancer. Squamous cell carcinomas account for about 75% of invasive cervical carcinoma cases. The main treatment methods for cervical cancer patients include surgery or concurrent radiotherapy and chemotherapy regimens, such as chemotherapy concurrent with radiotherapy and afterloading brachytherapy (Small et al., 2017). Clinical trials including the adoptive T-cell therapy, checkpoint inhibitors, and human papillomavirus (HPV) vaccines have shown satisfactory results. Based on these treatments, 3-years local control rate of patients with early-stage CESC is above 87%. The 5-years survival rate is 16.5%, and the median survival time is only 8–13 months for metastatic cervical cancer because of no standard treatment due to their heterogeneous manifestations (van Meir et al., 2014).

In most cases, the patient has already advanced to the moderate or late stages when first diagnosed. Therefore, invasion and metastasis are the major characteristics and causes of mortality in patients with advanced cervical cancer. *Cancer* cells acquire metastatic potential and gain motility mostly through the epithelial–mesenchymal transition (EMT) process. EMT is a cellular process characterized by the transformation from an epithelial phenotype to a mesenchymal phenotype (Nieto et al., 2016). This process plays a significant role in cancer initiation, invasion, metastasis, tumor immunosuppression, and immune evasion (Nieto et al., 2016; Jiang and Zhan, 2020). Targets associated with EMT regulation preventing cancer invasion and metastasis could be found to improve the prognosis of cervical carcinoma patients.

Novel increasing evidence showed that the process of EMT could be promoted or inhibited by autophagy (Catalano et al., 2015; Dash et al., 2018; Daskalaki et al., 2018; Wang et al., 2019; Wu et al., 2019; Liu et al., 2020). Autophagy is a catabolic procedure involved in maintaining homeostasis by metabolizing intracellular proteins and organelles under various conditions of cellular stress (Dikic and Elazar, 2018). There was a complicated regulatory network between the regulation of EMT and autophagy. EMT-involved oncogenic signal proteins such as SNAI1, SLUG, ZEB1/2, and NOTCH1 were functionally related to autophagy (Zada et al., 2021). Autophagy plays a tumor-inhibitory effect in the initial stages of carcinogenesis and a tumor-promotion function during the cancer progression (White, 2012). By its controversial role in tumor, the acquisition of EMT phenotype required autophagy activation during cancer progression and could be inhibited by autophagy inducers in the early stage of tumor formation (Hu et al., 2018; Wang et al., 2020). ARGs, especially the core mammalian autophagy proteins including ATG2–10, 12–14, 16–18, were a series of functional proteins implicated in the regulation of autophagy. However, the role of specific ARGs during the EMT process of cervical cancer has not yet been reported. DNA methylation is an epigenetic modification process that affects the expression of many genes, contributing to genome stability and the regulation of gene transcription (Moore et al., 2013). Methylation genes such as cadherin 1 (CDH1), death-associated protein kinase 1 (DAPK1), telomerase reverse transcriptase (TERT), and cell adhesion molecule 1 (CADM1) have been found as the highest methylation frequency genes (Wentzensen et al., 2009) and identified as promising markers for prognostic prediction of cervical cancer patients (Overmeer et al., 2011; Abudukadeer et al., 2012; Sun et al., 2015). Although some ARGs have been identified to prospectively predict the prognosis in patients with squamous cell cervical cancer (Chen et al., 2020; Shi et al., 2020), the DNA methylation of ARGs has not been analyzed in the prediction of cervical cancer prognosis.

In this paper, we aimed to find the clinical prognostic values of essential ARGs in cervical cancer based on the TCGA database. We found that a high level of ATG5 expression was an important poor prognostic factor in cervical cancer. In addition, further studies were performed to verify the role and the potential mechanisms of ATG5 in promoting migration and invasion in cervical cancer cell lines. Our results suggested that ATG5 acted as a poor prognostic marker and may be applied as a potential target for reversing the invasion and metastasis in cervical cancer.

## Materials and Methods

### Data Source and Preprocessing

RNA-seq reads data, methylation 450 k data, and clinical data of patients with cervical carcinoma from TCGA were acquired from Broad Institute Firehose (https://xenabrowser.net/datapages/). ARGs were obtained from the Human Autophagy database (HADb http://www.autophagy.lu/project.html). Samples without clinical survival status follow-up data were excluded from the study. Overall, a total of 296 CESC samples were enrolled in the present study. The 31 core ARGs involved in the further analysis are described in Table 1. These ARGs, identified as important regulators of autophagy, were essential for autophagosome formation and were involved in the further result analysis. Rstudio and R programming(4.03) were the principal tools for analyzing data throughout the study.

**TABLE 1:** Autophagy-related genes and their function in autophagy.

Genes		Autophagy-related function description	References
Human	Yeast	—	—
ATG2A/B	Atg2	Is part of ATG2–WIPI complexes involved in transferring lipid to the endoplasmic reticulum and the phagophore formation	Otomo and Maeda, (2019); Tang et al. (2017)
ATG3	Atg3	An E2-like enzyme that conjugates Atg8 with phosphatidylethanolamine; is essential for maintaining mitochondrial integrity	Besteiro et al. (2011); Yamaguchi et al. (2010)
ATG4A-D	Atg4	Cysteine protease mediates Atg8/LC3 lipid conjugation system	Agrotis et al. (2019); Zhang et al. (2016)
ATG5	Atg5	Participates in the formation of Atg12-Atg5/Atg16L1 (E3 ubiquitin ligase-like complex) and is required for autophagosome formation	Vij et al. (2016)
BECN1	Atg6	A component of the BECN1–PIK3C3–PIK3R4 complex and is indispensable for autophagy induction	Feng et al. (2014)
ATG7	Atg7	An E1-like enzyme that is essential for ATG12-conjugation and LC3-lipidation	Tanida et al. (2012)
MAP1LC3A/B/C; GABARAP; GABARAP L1–3	Atg8	Conjugated to PE by the two conjugation systems involving ATG7, ATG10, ATG3, ATG5, ATG12 and ATG16; act as autophagosome marker	Birgisdottir et al. (2013); Mizushima et al. (2011)
ATG9A/B	Atg9	The sole transmembrane protein driving autophagosomal membrane expansion	Matoba et al. (2020)
ATG10	Atg10	A protein-conjugating enzyme in the ATG12–ATG5 conjugation	Phillips et al. (2008)
ATG12	Atg12	A ubiquitin-like modifier; forms a multimeric complex with Atg16 and Atg5	Hanada and Ohsumi, (2005)
ATG13	Atg13	Part of ULK–Atg13–FIP200 complexes involved in autophagosome formation	Jung et al. (2009)
ATG14	Atg14	Mediates fusion of autophagosomes to endolysosomes; a component of the BECN1-ATG14-containing phosphatidylinositol 3-kinase complex	Diao et al. (2015); Zhao et al. (2020)
ATG16L1/L2	Atg16	Participates in ubiquitin-like protein conjugation systems that are important for autophagosome formation and membrane elongation	Mesquita et al. (2017); Xiong et al. (2018)
ATG101	—	Component of the ULK1 complex; an accessory protein involved in ATG13 stabilization; bridges the ULK1 and PtdIns3K complexes	Kim et al. (2018); Zachari and Ganley, (2017)
ULK1/2	—	Comprises of the ULK–ATG13–FIP200–ATG101 complex involved in autophagy induction	Zachari and Ganley, (2017)
RB1CC1	Atg17	Parts of the ULK1 complex (ULK1, RB1CC1, ATG13, and ATG101) involved in autophagy initiation	Hurley and Young, (2017)
WIPI1/2	Atg18	Recognizes and decodes the PtdIns3P signal; facilitates the ATG12-5/ATG16 conjugates LC3 to phosphatidylethanolamine	Dooley et al. (2014); Proikas-Cezanne et al. (2015)

### Survival Analysis of ARGs in CESC

The optimal cutoff values of ARGs based on the expression Fragments Per kilobase per Million mapped reads (FPKM) value or methylation Beta-value, the survival time, and the survival status were identified by the “surv_cutpoint” function in “survminer” package as described before (Wang et al., 2021). The cutoff value of the OS significant genes is listed in Supplementary Figure S1. The patients with relative values above or below the optimal cutoff were considered as high or low groups for each gene expression level, methylation level, or EMT score. Kaplan–Meier (KM) survival analysis with log-rank test was then used for investigating the OS difference between the abovementioned high and low groups. The correlation between the OS characteristics of CESC patients and each ARG expression was explored by a univariate Cox regression analysis. A multivariate Cox regression analysis was performed to investigate whether each ARG is an independent OS influence factor.

The patients with a relative value above or below the optimal cutoff were considered as high or low group for each gene expression level, methylation level, or EMT score. KM survival analysis with log-rank test was then used for investigating the overall survival (OS) difference between the abovementioned high and low groups.

### EMT Score Evaluation

By “GSVA” R package, the EMT score for evaluating the EMT status of each CESC patient was quantified by single-sample gene-set enrichment analysis (ssGSEA) based on previously reported EMT signature genes (Choi et al., 2010; Hanzelmann et al., 2013). The correlation between ATG5 and EMT score or between ATG5 and EMT signature related genes is calculated by Pearson’s correlation coefficient and plotted in heatmap by the “circlize” R package. The EMT score density between normal and tumor tissues and the survival difference between high and low groups were also analyzed.

### ATG5 Co-Expression Network Selection and Gene Functional Enrichment Analysis

The genes co-expressed with ATG5 in CESC were screened according to the Pearson correlation coefficient (|cor| > 0.3, *p* < 0.05) and plotted in the volcano plot by the “ggplot2” package. Heatmaps of the top 50 negative or the top 50 positive ATG5 expression correlated genes were plotted by the “pheatmap” R package. Metascape (Zhou et al., 2019) was employed to gain insights into the biological functions of those co-expressed genes of ATG5.

### Identification Prognostic Methylation Sites of ATG5

The methylation site information of ATG5 in TCGA CESC methylation data based on Illumina Infinium Human Methylation450 BeadChip platform was mapped by an annotation file retrieved from the hg19 GPL16304 legacy annotation file (Price et al., 2013). The methylation level of each site in ATG5 was grouped into low and high by the survminer package as mentioned above. The OS significant associated sites were screened through KM survival analysis. Besides, MEXPRESS was used for visualizing expression, DNA methylation, and clinical parameters based on TCGA data (http://mexpress.be). The correlation between the methylation level of each site of ATG5 and the expression of ATG5 is calculated by the “corrplot” package (Wei and Simko, 2021).

### Gene Mutation Analysis of ARGs in CESC

Waterfall plots of ARGs mutation were produced by the oncoplot function in the “maftools” R package (Mayakonda et al., 2018) to visualize the mutation status such as classification and frequency of mutation types, frequency of variant types, and frequency of SNV classes for CESC samples. The mutational exclusion and co-occurrence mutations among those ARGs were analyzed by Fisher’s exact test (Mayakonda et al., 2018). The variant allele frequency (VAF) distribution of ARGs was also plotted.

### Validation of the Prognosis Performance for Different Clinical Features of CESC

ALL CESC patients were grouped based on different clinical staging information (pathological—T/N/M, histologic—Grade/Stage) or the age at diagnosis (<45 years, 45–69 years, and >69 years). Subtypes of CESC were grouped into squamous cell neoplasms or adenocarcinoma based on the clinical data. The OS difference between the high- or low-ATG5 expression groups was evaluated in KM survival analysis.

### Cell Lines

Hela, Ca Ski (human cervical cancer cell lines), and PANC-1 cells (pancreatic cancer cell line) were purchased from the American Type Culture Collection (ATCC, Manassas, VA, United States). Hela cells were incubated in DMEM supplemented with 10% FBS (Gibco, Grand Island, NY). Ca Ski and PANC-1 cells were incubated in RPMI 1640 containing 10% FBS (Gibco, Grand Island, NY). PANC-1 cells were used as a positive control for ATG5 expression (Yang et al., 2015). Cells are cultured in an incubator at 37°C with 5% CO_2_.

### Cell Transfection

According to the manufacturer’s instructions (GeneChem Corporation, Shanghai, China), Hela and Ca Ski cells were transfected with the recombinant lentiviral vector LV-ATG5-RNAi labeled with green fluorescent to establish ATG5-knockdown cells and named as KD. In brief, the cells were digested at the logarithmic growth phase, then resuspended in the complete medium at a concentration of 3–5 ×10^4^ cells/ml, and seeded in six-well plates. At 20% confluence, cells were transfected with the recombinant lentivirus for 16 h. Subsequently, a conditioned medium containing transfection reagent was refreshed. At 72 h post-infection, the positive expression rate of GFP was used to evaluate the transfection efficiency under a fluorescence microscope (Olympus Corporation, Tokyo, Japan). siRNAs specific for ATG5 were obtained from GenePharma (Shanghai, China), and the detailed sequences were as follows: ATG5-RNAi (98917-1) sense: CCTTTCATTCAGAAGCT GTTT, ATG5-RNAi (98918-1) sense: CCTGAACAGAA TCATCCTTAA, ATG5-RNAi (98919-1) sense: GAT​TCA​TGG​AAT​TGA​GCC​AAT, NC-RNAi sense: TTC​TCC​GAA​CGT​GTC​ACG​T.

### Quantitative Real-Time Polymerase Chain Reaction (qRT-PCR)

Cells were collected and lysed by Trizol reagent (Invitrogen, Carlsbad, CA). Next, cDNA was obtained by using M-MLV reverse transcriptase (Promega, Madison, WA). Using a LightCycler 480 II RT-PCR System (Roche, Basel, Switzerland), qRT-PCR was performed as follows: 95°C for 30 s, and then 40 cycles of 95°C for 5 s and 60°C for 30 s. The sequences of primers amplified in this study were as follows: ATG5: forward 5′-AAGAGTGTTTATTCG TCGGT-3′, reverse 5′-ATC​ACA​GCT​TAG​TGT​TCC​CT-3′. ACTB: forward 5′-GCG​TGA​CAT​TAA​GGA​GAA​GC-3′, reverse 5′-CCACGTCACA CTTCATGATGG-3′. Beta-actin (ACTB) gene was used as an internal reference.

### Western Blot Assay

In short, cells were harvested and washed with PBS twice, and then lysed in RIPA buffer (Beyotime, Shanghai, China) on ice for 15 min. Then, BCA Protein Assay Kit (Beyotime, Shanghai, China) was used to detect the protein concentration in the supernatant from crushed cells by ultrasonication. The final concentration was adjusted to 2 μg/μl by fresh RIPA solution, followed by protein denaturation. 10% SDS-PAGE was applied to separate the protein samples and then transferred onto PVDF membranes (Millipore, Burlington, MA). After that, membranes were incubated with TBST containing 5% skim milk to block non-specific antigens. Next, the membranes were incubated with primary antibodies and HRP-conjugated second antibodies in turn. Finally, the protein bands were visualized by the electrochemiluminescence (ECL) detection system. The primary antibodies used include mouse anti-ATG5 antibody (1:500, sc-133158, Santacruz, Santa Cruz, CA), mouse anti-ERK antibody (1:2,000, #9107,CST, Danvers, MA, United States), rabbit anti-NFκB p65 antibody (1:3,000, #8242, CST, Danvers, MA, United States), rabbit anti-mTOR antibody (1:1,000, #2983, CST, Danvers, MA, United States), rabbit anti-*p*-ERK antibody (1:1,000, #4376, CST, Danvers, MA, United States), rabbit anti-*p*-NFκB p65 antibody (1:500, ab76302, Abcam, Cambridge, MA, United States), rabbit anti-*p*-mTOR antibody (1:500, ab109268, Abcam, Cambridge, MA, United States), rabbit anti-P38 antibody (1:3,000, #8690, CST, Danvers, MA, United States), rabbit anti-AKT antibody (1:500, #4691, CST, Danvers, MA, United States), mouse anti-E-Cadherin antibody (1:500, #14472, CST, Danvers, MA, United States), rabbit anti-p-P38 antibody (1:5,000, #4631, CST, Danvers, MA, United States), rabbit anti-*p*-AKT antibody (1:1,000, #4060, CST, Danvers, MA, United States), rabbit anti-N-Cadherin antibody (1:500, ab18203, Abcam, Cambridge, MA, United States), mouse anti-Twist antibody (1:100, ab50887, Abcam, Cambridge, MA, United States), rabbit anti-c-Myc antibody (1:1,000, ab32072, Abcam, Cambridge, MA, United States), rabbit anti-Snail antibody (1:500, #3879, CST, Danvers, MA, United States), rabbit anti-β-Catenin antibody (1:2,000, #8480, CST, Danvers, MA, United States), rabbit anti-Slug antibody (1:500, #9585, CST, Danvers, MA, United States), rabbit anti-p-β-Catenin antibody (1:500, #2009, CST, Danvers, MA, United States), rabbit anti-MMP-2 antibody (1:500, #40994, CST, Danvers, MA, United States), mouse anti-Fibronectin antibody (1:500, MAB 1918, R&D Systems, Minneapolis, MN, United States), rabbit anti-Vimentin antibody (1:500, #5741, CST, Danvers, MA, United States), and rabbit anti-MMP-9 antibody (1:500, #13667, CST, Danvers, MA, United States). Mouse anti-β-actin (1:5,000, sc-69879, Santacruz, Santa Cruz, CA) and mouse anti-GAPDH (1:10,000, ab37168, Abcam, Cambridge, MA, United States) were used as an internal control.

### Cell Migration and Invasion Assays

Cells were seeded in the upper chamber coated with (invasion) or without (migration) Matrigel (Corning Costar, Cambridge, MA, United States) at a density of 1 × 10^5^ cells/well. In invasion assay, serum-free medium was added into the lower chamber. In the migration assay, a medium containing 30% FBS was added to the lower chamber. The noninvasive/non-migrated cells were removed by a cotton swab after 48-h incubation. Then, the cells were fixed in 4% paraformaldehyde for 0.5 h and stained with 0.5% crystal violet solution, followed by taking photographs through the microscope.

### Statistical Analysis

All molecular biology assays were repeated in triplicate. Experimental data are presented as the mean ± SD. R (version 4.03) and Rstudio software were applied to perform statistical analysis. The differences between the groups were analyzed *via* Student’s *t*-test and Mann–Whitney *U* test. Univariate and multivariate Cox regression analysis were performed to identify the factors influencing prognosis. Survival analysis was done using the KM method and the log-rank test. *p* < 0.05 was designated as statistically significant.

## Results

### Baseline Information of the CESC Patients

A total of 309 sequencing data and 312 methylation data in TCGA CESC cohort were obtained from the TCGA data. The median age of those 306 participants was 46.57 years (min 20.95 years and max 89.04 years). The median follow-up time for CESC patients with survival information was 1.87 years (range 0–17.55 years), and among them, 225 were alive and 74 were dead during the follow-up time.

### Prognosis of ARGs in CESC

**FIGURE 1:**
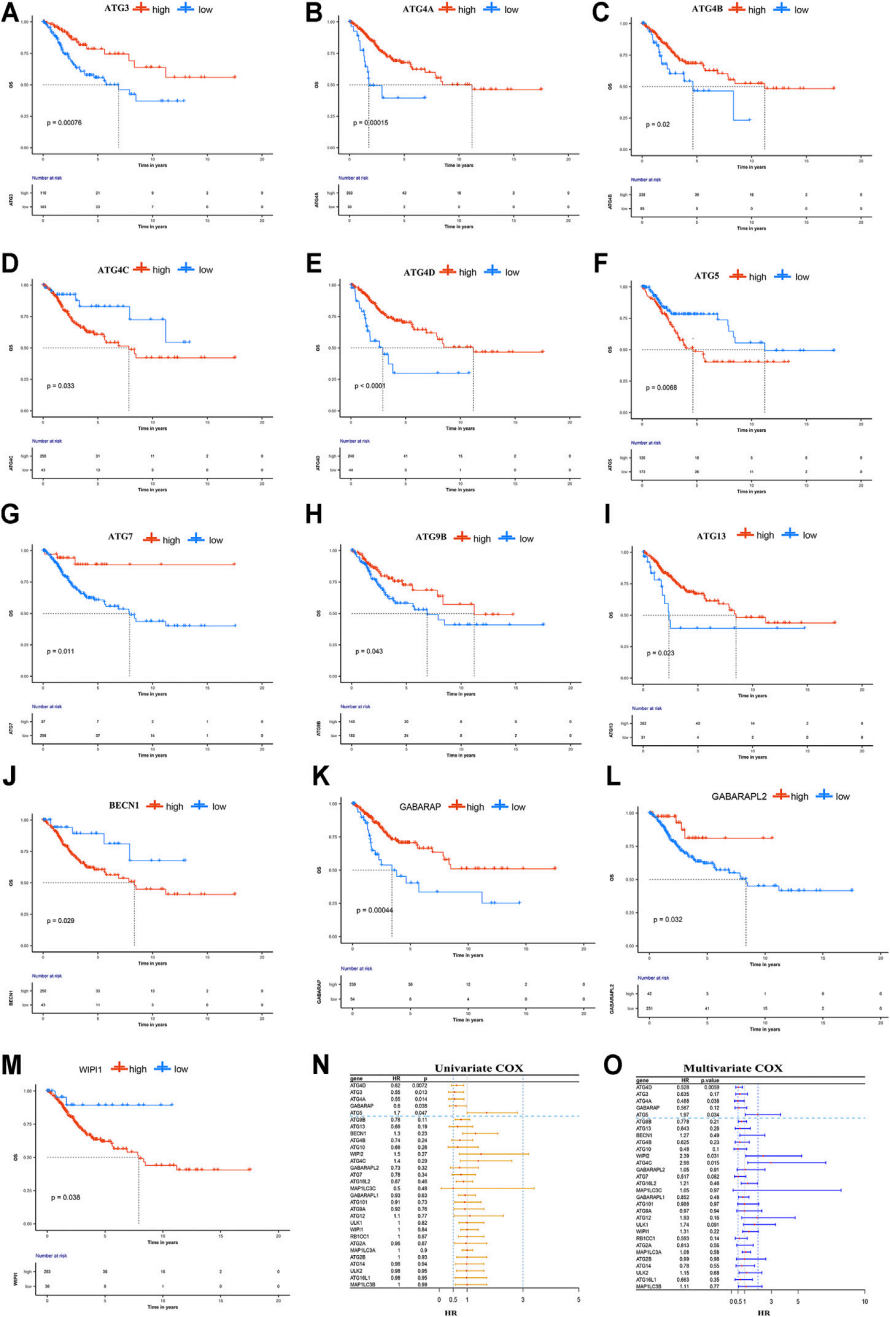
Overall survival analysis on ARGs in cervical cancer. The expression of **(A)** ATG3 **(B)** ATG4A **(C)** ATG4B **(E)**ATG4D (G) ATG7 **(H)** ATG9B **(I)** ATG13 **(K)**GABARAP, and **(L)** GABARAPL2 were associated positively with OS, while increased expression of **(D)** ATG4C **(F)** ATG5 **(J)** BECN1, and **(M)** WIPI1 negatively correlated with OS. Figure **(N, O)** According to the forest plot for the Univariate and Multivariate Cox regression analysis results, ATG5 was a risk prognostic factor, while ATG4D, GABARAP, ATG4A, and ATG3 were favorable prognostic factors in CESC patients. And, ATG5, ATG4D, and ATG4A were independent prognostic factors.

**FIGURE 2:**
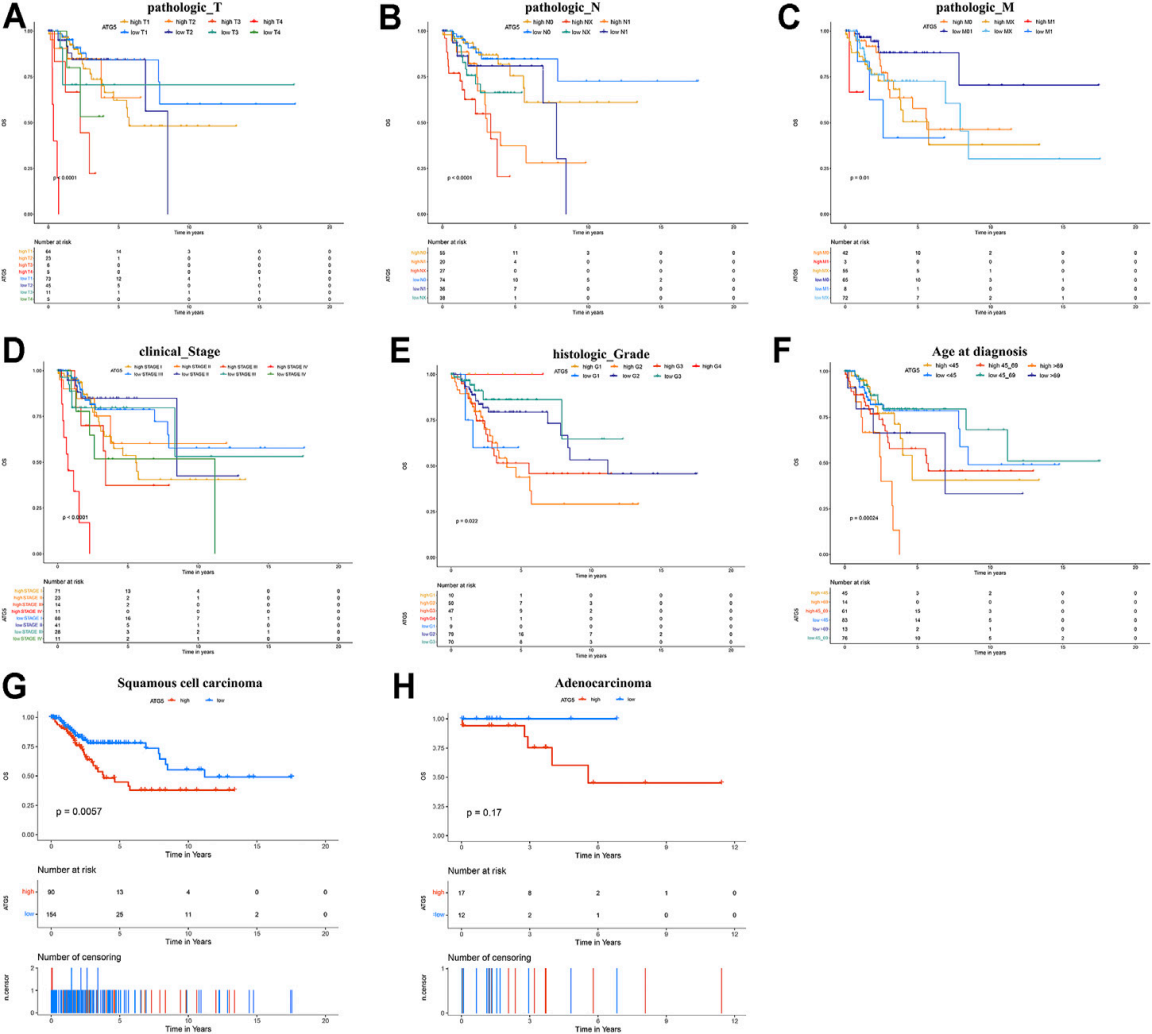
Kaplan-Meier survival analysis of ATG5 compared with different clinical parameters. By comparison, high expression of ATG5 in cervical patients with most different **(A)** T stage **(B)** N stage **(C)** M stage **(D)** FIGO stage **(E)** pathological grade, and **(F)** Ages at diagnosis showed shorter median OS time; Cervical patients with **(G)** Cervical squamous cell carcinoma, and **(H)** Cervical adenocarcinoma had shorter OS in ATG5 overexpression group.

High expression levels of ATG4C, ATG5, BECN1, and WIPI1 were associated with poor prognosis in CESC, while CESC patients with overexpression of ATG3, ATG4A, ATG4B, ATG4D, ATG7, ATG9B, ATG13, GABARAP, and GABARAPL2 have longer OS and higher 5-years survival rate (Figure 1). Furthermore, the univariate Cox regression results indicated that ATG5, ATG4D, GABARAP, ATG4A, and ATG3 were significantly associated with OS (Figure 1N). ATG5 was identified as a risk prognostic predictor in CESC patients (HR 1.7; 95% CI, 1.0–2.8, *p* = 0.047), while another four ARGs were identified as favorable prognostic factors (HR < 1, *p* < 0.05). Subsequently, the multivariate Cox results showed that ATG5, ATG4D, and ATG4A were independent prognostic factors affecting OS of patients with CESC (Figure 1O). Among them, high expression of ATG5 or low expressions of ATG4D and ATG4A were associated with unfavorable prognosis. These results indicated that ATG5 among these ARGs was the most harmful factor affecting the prognosis of CESC patients. According to the KM analysis, the 5-years survival rate for high- and low-expression groups of ATG5 is 0.486 (0.375–0.631) and 0.782 (0.708–0.863) respectively. Furthermore, KM survival analysis was also performed to show that ATG5 expression could effectively predict the prognosis of patients with CESC with different clinical stages, pathological grades, and ages at diagnosis (Figures 2A–F). For patients with cervical squamous cell carcinoma, patients with ATG5 overexpression have significantly shorter OS than the ATG5 down-expression group (Figure 2G). For cervical adenocarcinoma, due to the limited sample size of patients included in the analysis, the *p*-value of the km survival curve is not significant, but patients with ATG5 down-expression showed a trend towards a longer survival with no death during the follow-up times (Figure 2H).

### Co-Expression Genes Correlated With ATG5 in CESC

**FIGURE 3:**
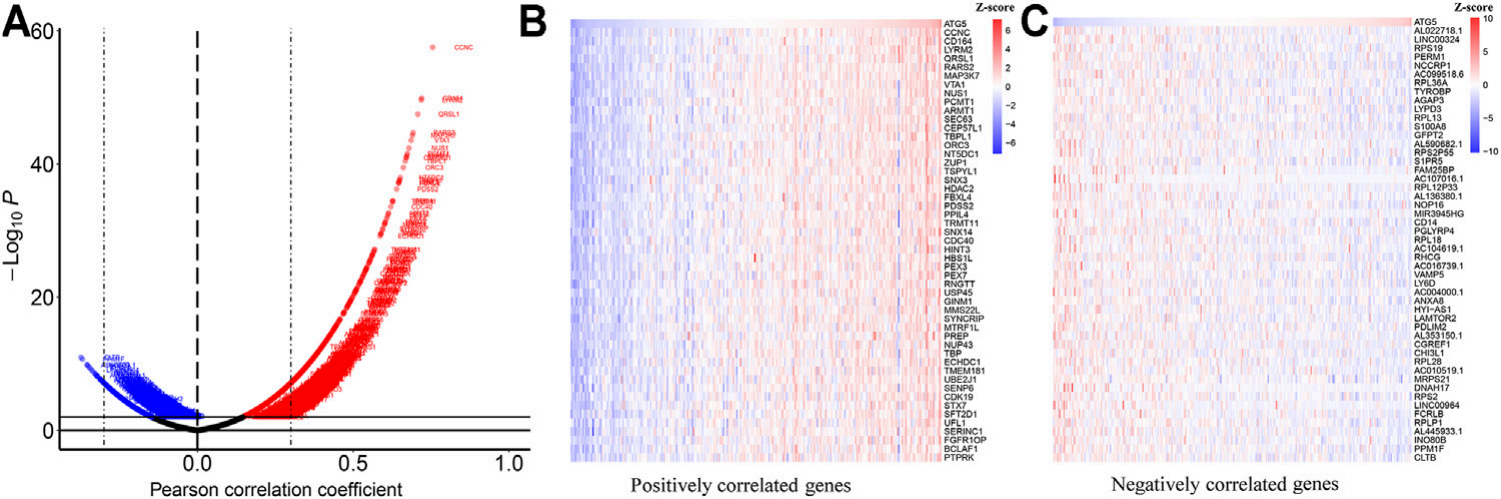
Co-expression genes correlated with ATG5 in cervical cancer (LinkedOmics) **(A)** Pearson test was applied to analyze the correlation between ATG5 and differentially expressed genes in Cervical cancer. Blue dots (R < −0.3, *p* < 0.01) represent as negatively correlated genes, while red dots (R > 0.3, *p* < 0.01) represent as positively correlated genes **(B)** Heat map showed the top 50 significant genes positively correlated with ATG5 **(C)** Heat map showed the top 50 significant genes negatively correlated with ATG5 (The expression of each in each heatmap is normalized with Z-score).

**FIGURE 4:**
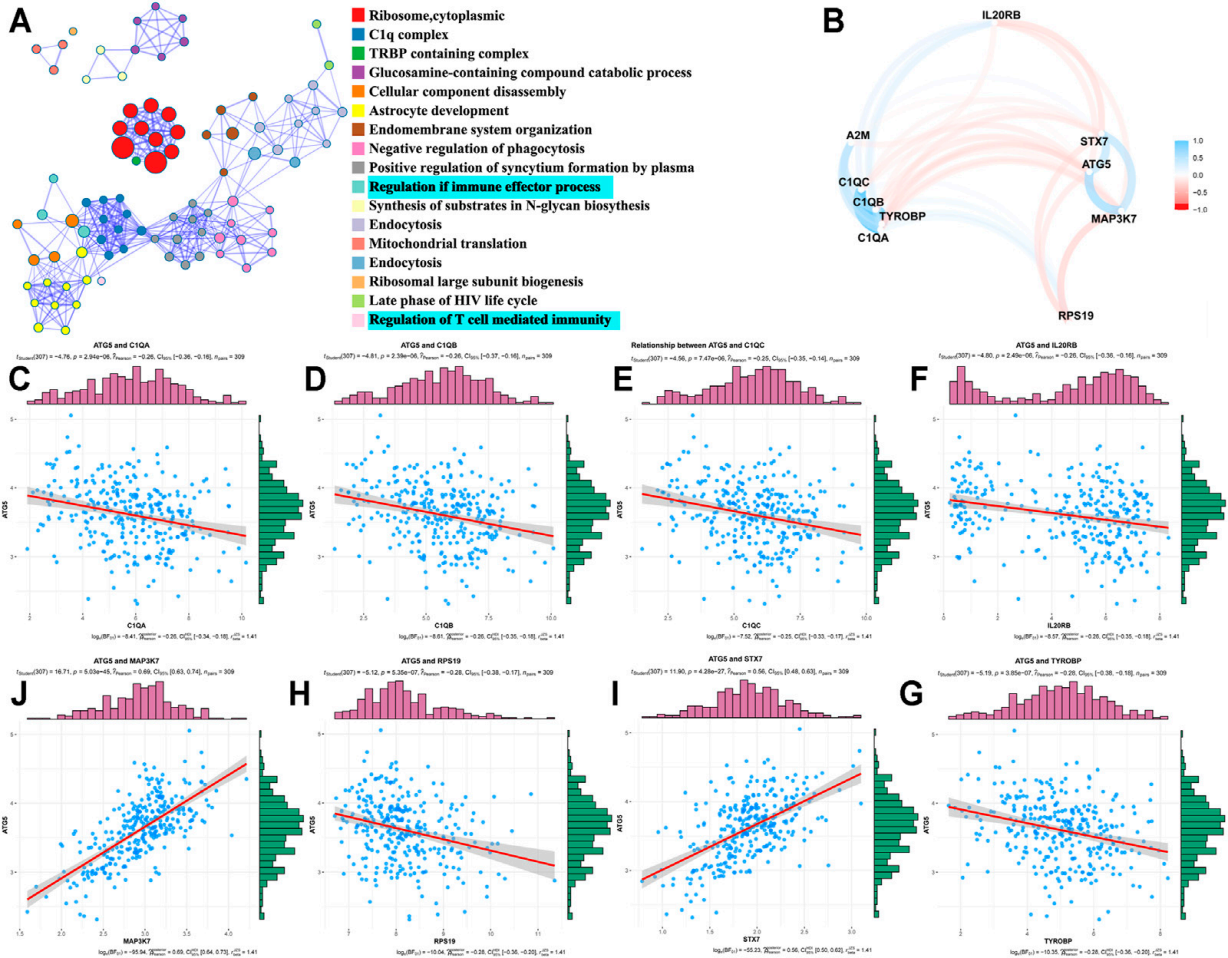
GO analysis and correlation analysis **(A)** GO analysis revealed the biological processes and molecular functions involved in the top 100 ATG5-related co-expressed genes by “metascape” data **(B)** The correlation between ATG5 and co-expressed genes involved in the regulation of the immune effector process in cervical cancer patients. Blue lines represent as negatively correlated genes, while red lines represent as positively correlated genes **(C-J)** Among those regulation of the immune effector process genes, ATG5 expression was significantly negatively correlated with MAP3K7, STX7 and significantly positively related to A2M, C1QA, C1QB, C1QC, RPS19, TYROBP, and IL20RB.

**FIGURE 5:**
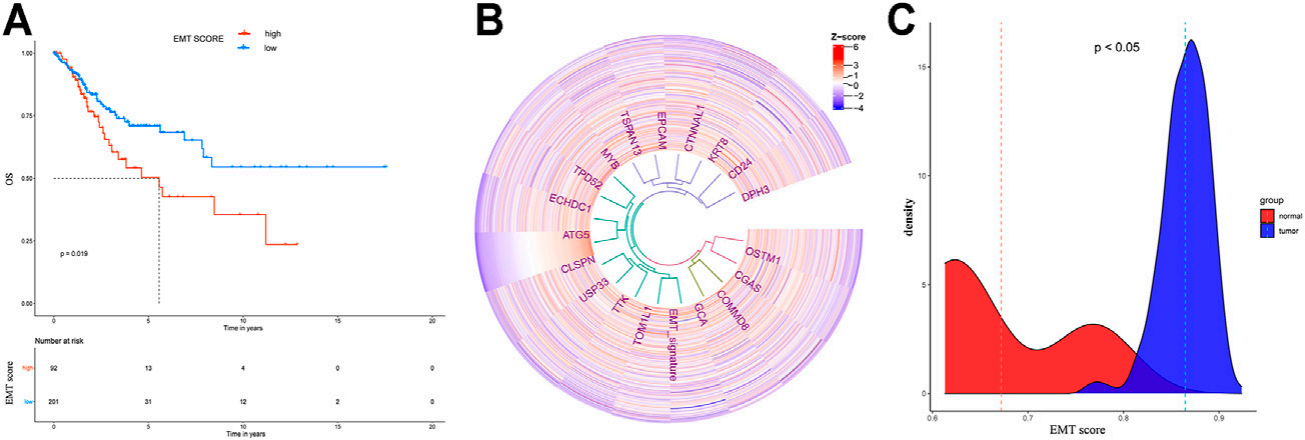
The correlation between ATG5 and co-expressed genes involved in the regulation of EMT in cervical cancer patients. A. the Kaplan-Meier survival plot showed that the median OS time of low EMT score patients was significant longer than high EMT score patients. B. ATG5 showed co-expressed ship with genes involved in the regulation of EMT in cervical cancer patients. C. The EMT signature showed significant difference in cervical normal and cancer tissues.

Based on the TCGA database, we analyzed the co-expressed genes of ATG5 in 304 CESC patients through the Pearson correlation coefficient. As shown in Figure 3A, red dots represented 5,666 genes positively associated with ATG5, while blue dots represented 2,114 genes negatively associated with ATG5. The heat map was used to show the top 50 genes positively or negatively correlating with ATG5 (Figures 3B,C). Next, GO enrichment analysis was performed to investigate the biological pathway of the top ATG5-related co-expressed genes. ATG5 co-expressed genes are correlated with ribosome, cytoplasmic, C1 complex, TRBP-containing complex, glucosamine-containing compound catabolic process, and so on. There are concerns that ATG5 also participated in the regulation of the immune effector process and regulation of T-cell-mediated immunity (Figure 4A). We further found that the ATG5 co-expressed genes involved in the regulation of the immune effector process were A2M, C1QA, C1QB, C1QC, RPS19, MAP3K7, TYROBP, STX7, and IL20RB. MAP3K7, STX7, and IL20RB were involved in the regulation of T-cell-mediated immunity. Moreover, ATG5 expression was inversely associated with MAP3K7 and STX7 and positively related to A2M, C1QA, C1QB, C1QC, RPS19, TYROBP, and IL20RB (Figures 4B–J). Furthermore, it was important to investigate the relation between ATG5 and EMT-related gene signature. The result is represented in Figure 5. We found that ATG5 was significantly correlated with EMT scores quantified by ssGSEA and EMT signature-related genes (Choi et al., 2010) such as DPH3, CD24, KRT8, CTNNAL1, EPCAM, TSPAN13, MYB, TPD52, ECHDC1, CLSPN, USP33, TTK, TOM1L1, GCA, COMMD8, CGAS, OSTM1, and EMT score. Tumor tissues had higher EMT scores than normal tissues, and a high EMT score predicted shorter survival time for CESC patients.

### Genomic Alterations and Methylation of ARGs in CESC

**FIGURE 6:**
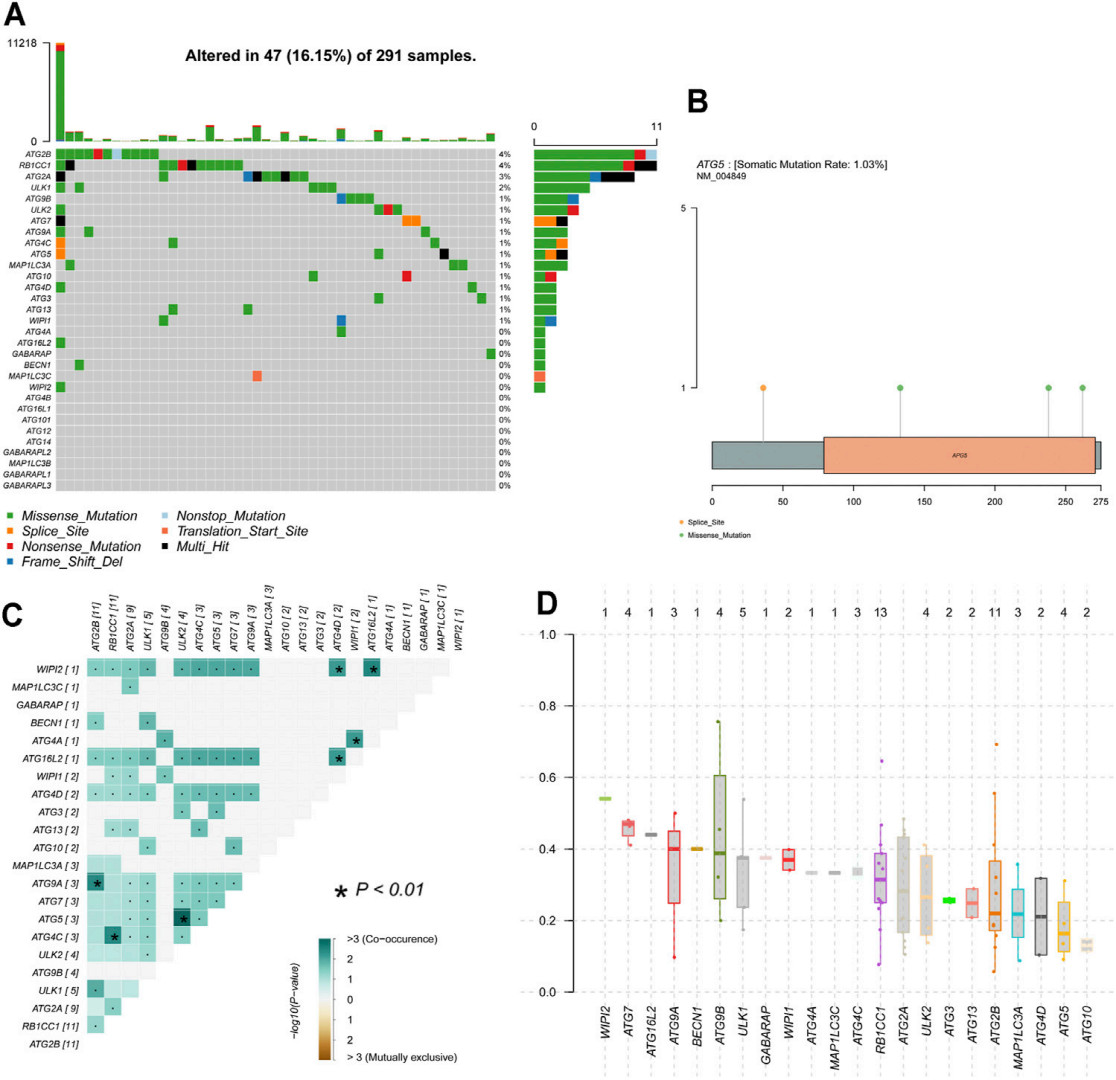
Mutation data analysis of ARGs in CESC **(A)** Waterfall plots of ARGs mutation results show that ATG2B and RB1CC1 are the most frequently mutated genes. A total of 16 genes has a mutation rate ≥1% **(B)** mutation type and domain of ATG5 **(C)** The co-occurrence of ARGs mutation in cervical cancer. Dot represents as no statistical significance, *p < 0.01. Green means co-occurrence **(D)** The variant allele frequency (VAF) distribution of ARGs in CESC by plotvaf function in “maftools” package. Each dot in the boxplot represents a variant. The total number of variants is list on the top of each box.

**FIGURE 7:**
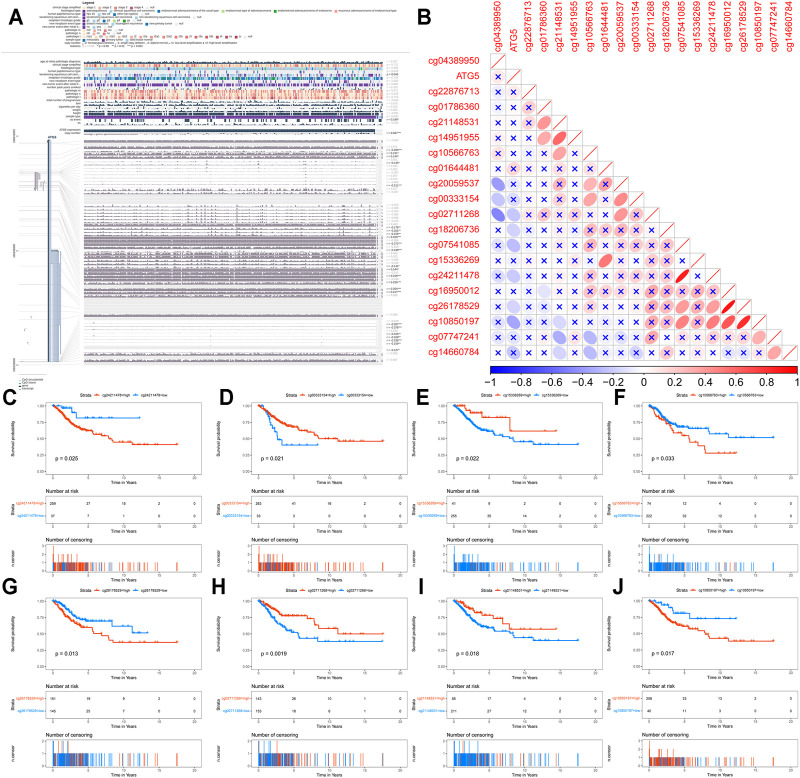
Genomic methylation of ARGs in CESC **(A)** The methylation of ATG5 gene was significantly correlated with the OS in cervical cancer patients **(B)** The correlation between methylation sites of ATG5 and ATG5 expression in cervical cancer patients. Purple represents negative correlation; red represents positive correlation; “×” represents no statistical significance **(C-J)** Kaplan-Meier survival analysis showed the methylation sites of ATG5 significantly related to OS in cervical cancer patients (*p* < 0.05). cg00333154, cg15336269, cg02711268, and cg21148531 located in ATG5 were beneficial for OS, while cg24211478, cg10566763, cg26178529, and cg10850197 located in ATG5 were harmful for OS.

Genetic alteration results showed that 16.15% of patients own mutations of these ARGs. Among these genes, a total of 16 genes have a mutation rate ≥1%, of which ATG2B and RB1CC1 are the most frequently mutated genes (4%). Missense and splice are the two most common types of mutations (Figure 6A). ATG5 was altered in 3 (1.03%) of the 291 CESC patients. Figure 6B shows the mutation type and domain of ATG5. Moreover, concomitant occurrence of ATG5 mutation and ULK2 mutation was found in CESC (Figure 6C). Compared with most mutated ARGs, ATG5 owns a relatively low VAF, except ATG10. MEXPRESS was used for visualizing expression, DNA methylation, and clinical parameters based on TCGA data, and showed the dramatic relationship between ATG5 methylation status and OS in CESC patients (Figure 7A). According to the annotation file of Methylation450 BeadChip platform, there are 19 methylation sites of ATG5, namely, cg24211478, cg14951955, cg00333154, cg07541085, cg15336269, cg07747241, cg01786360, cg16950012, cg22876713, cg10566763, cg26178529, cg01644481, cg02711268, cg14660784, cg18206,736, cg20059537, cg21148531, cg04389950, and cg10850197. As shown in Figure 7B, there was a negative correlation between ATG5 expression and the methylation sites cg02711268, cg18206,736, cg07541085, cg24211478, cg26178529, and cg10850197. The significant sites of those methylation sites in ATG5 on OS in CESC patients were shown in KM including beneficial OS relevance methylation sites such as cg00333154, cg15336269, cg02711268, and cg21148531 and the harmful OS-related sites such as cg24211478, cg10566763, cg26178529, and cg10850197 (Figures 7C–J).

### The Regulation of ATG5 Expression in Cervical Carcinoma Cells

**FIGURE 8:**
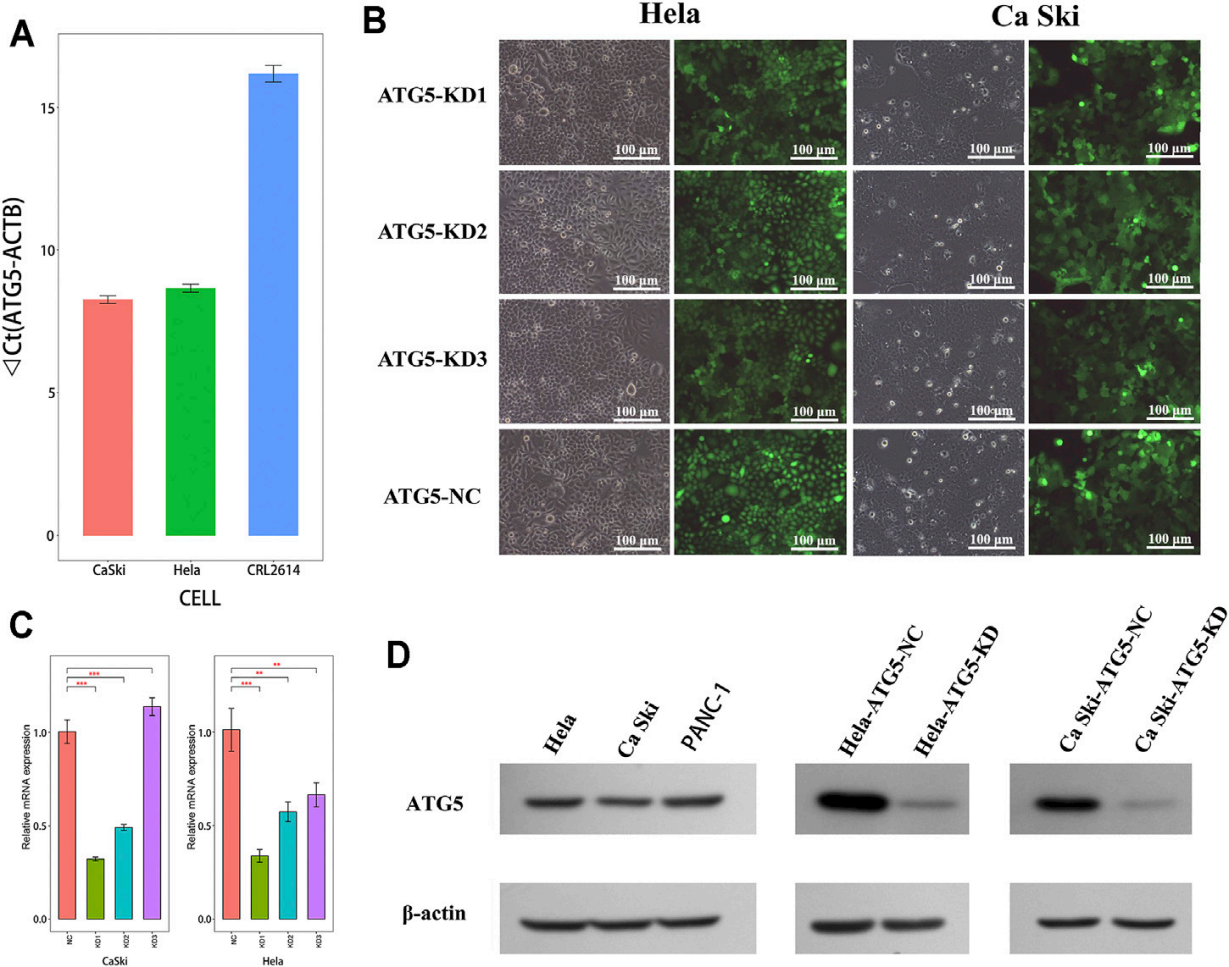
Expression of ATG5 in cervical cancer cell lines **(A)** The expression of ATG5 mRNA was detected in Hela and Ca Ski **(B)** After 72 h infection, Hela and Ca Ski cells with green fluorescence were the cells that transfected plasmid successfully. Magnification ×100 **(C)** mRNA expression of ATG5 was significantly inhibited by ATG5-RNAi compared with the control group. Error bars represent ±SD. *p < 0.05, **p < 0.01, ***p < 0.001 **(D)** Protein expression of ATG5 could be detected in Hela and Ca Ski, and was significantly downregulated by ATG5-RNAi in Hela-ATG5-KD and Ca Ski-ATG5-KD cells compared with the control group. PANC-1 was used as a positive control.

ATG5 mRNA showed higher expression in cervical cancer cell lines Hela and Ca Ski compared with the cervical epithelial cell line CRL2614 (Figure 8A). The recombinant lentiviral vector containing ATG5-RNAi labeled with green fluorescence was used to downregulate ATG5 expression of Hela and Ca Ski cells. Cells presented with green fluorescence indicated the success of transfection (Figure 8B). RT-qPCR showed that the levels of ATG5 mRNA were significantly downregulated in Hela-KD (knockdown) and Ca Ski-KD cells compared with NC (negative control) cells (Figure 8C, *p* < 0.01). In addition, the sequence with the highest knockdown efficiency was selected for use in the next study. The knockdown efficiency was further confirmed by Western blot (Figure 8D).

### Knockdown of ATG5 Impedes Cervical Cancer Cells Migration and Invasion

**FIGURE 9:**
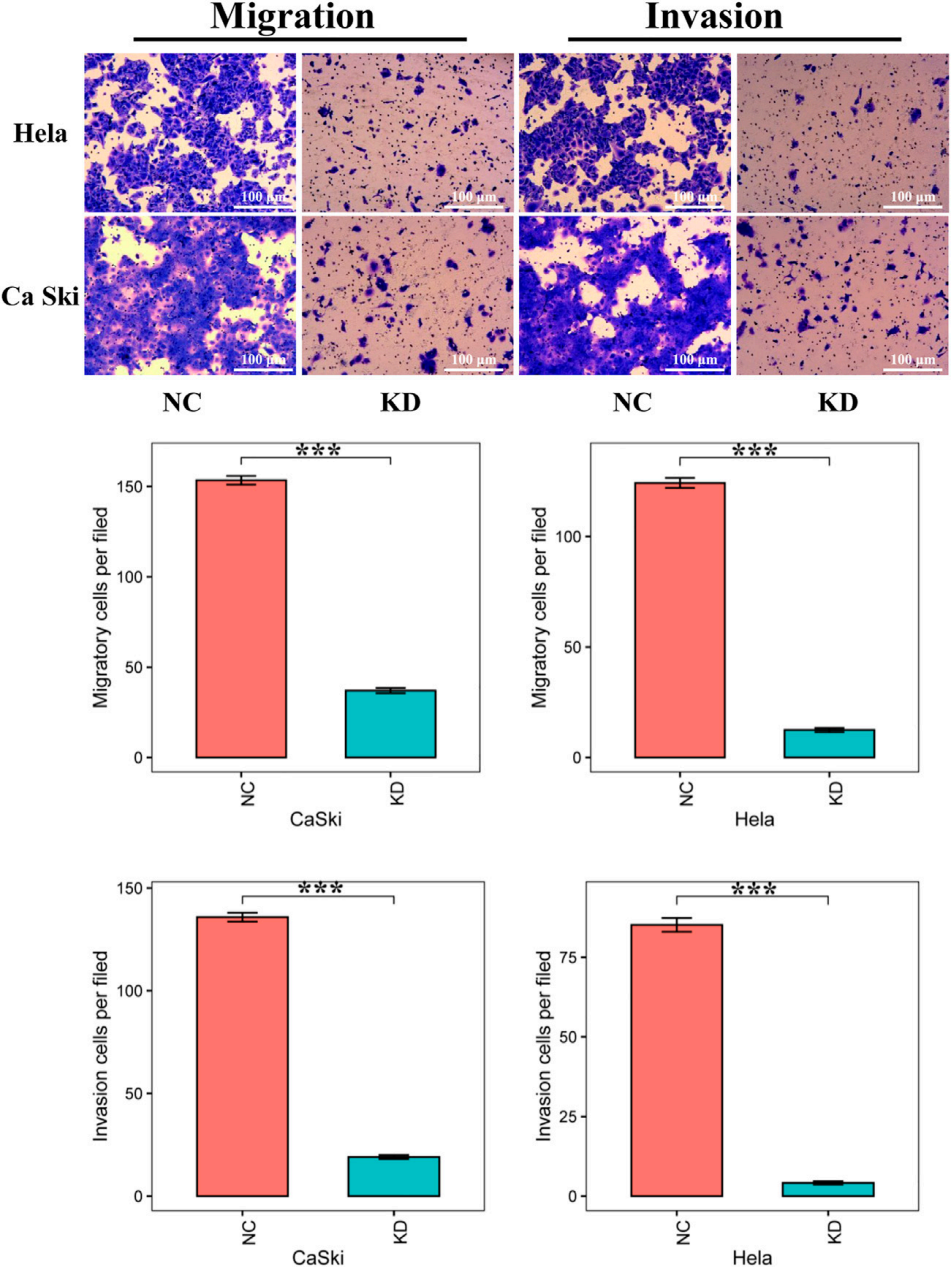
Down-regulation of ATG5 significantly decelerates cervical cancer cells migration and invasion. Magnification ×100. Error bars represent ±SD. ***p < 0.001.

By Transwell migration assay, ATG5 knockdown demonstrated a markedly negative effect on the migratory capacity of Hela and Ca Ski cells compared with NC (Figure 9). Subsequently, the role of ATG5 in invasion was assessed by a Transwell invasion assay. Likewise, the data of the Transwell invasion assay showed that downregulation of ATG5 dramatically suppressed the invasion in cervical cancer cells (Figure 9). In brief, these results exhibited the repression of ATG5 in the migration and invasion of cervical cancer cells *in vitro*.

### ATG5 Knockdown Reversed EMT of Cervical Cancer Cells

**FIGURE 10:**
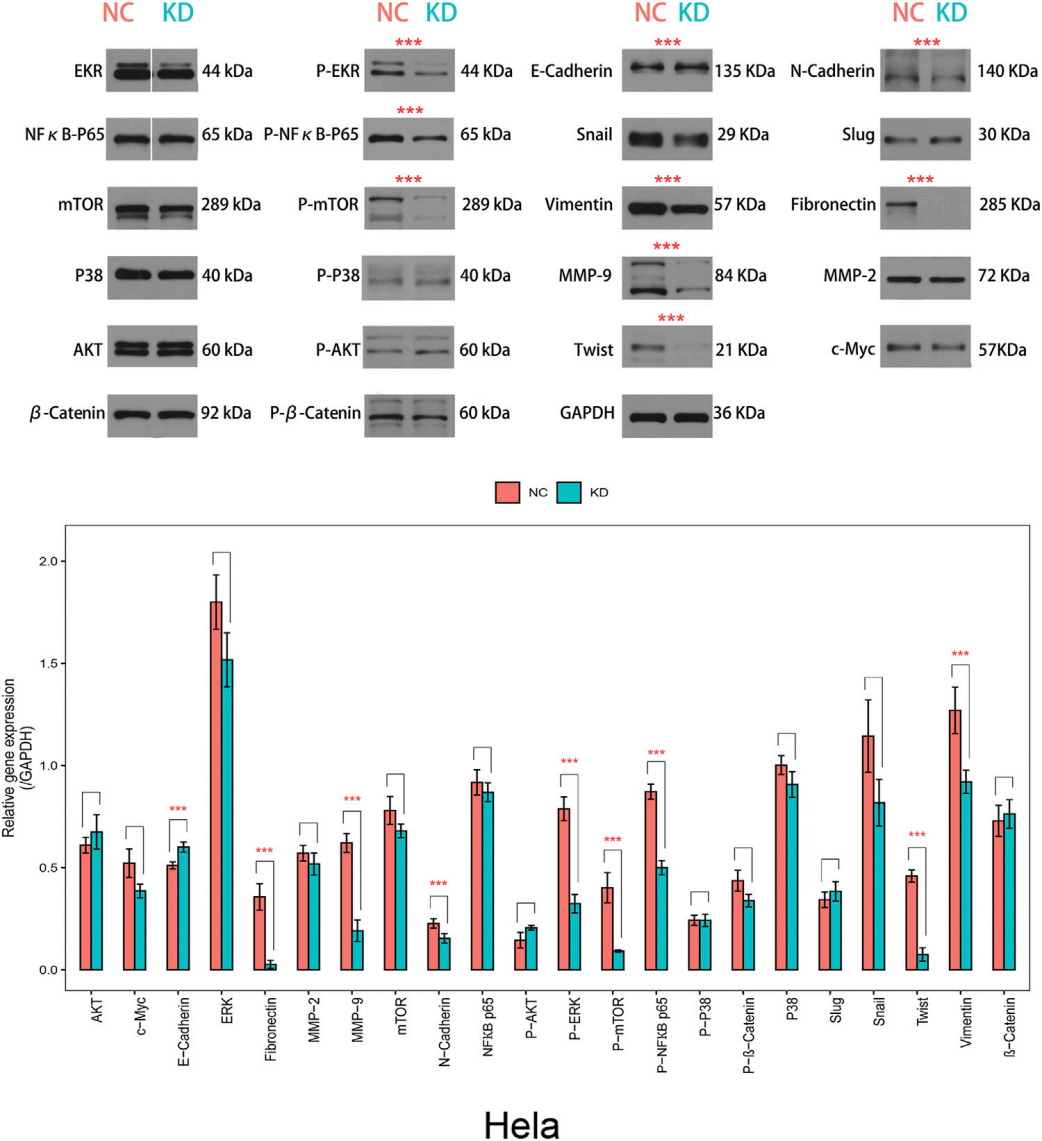
The effect of ATG5 knockdown on the protein expression of EMT-related genes. The expression of N-cadherin, Vimentin, P-ERK, P-NFκBp65, P-mTOR, MMP-9, and Twist was decreased in knockdown of ATG5 group (KD) compared to control group (NC), instead, E-cadherin expression was enhanced. Error bars represent ±SD. ***p < 0.001.

Further investigation found that knockdown of ATG5 in Hela cells downregulated expressions of interstitial phenotypic markers (N-cadherin and Vimentin), EMT signaling pathway-related proteins (P-ERK, P-NFκBp65, and P-mTOR), extracellular matrix metalloproteinases (MMP-9), CC-specific interstitial phenotypic marker (Fibronectin), and EMT-related transcription factors (Twist), but upregulated epithelial marker E-cadherin (Figure 10). However, other markers including NFκBp65, mTOR, P38, AKT, P-P38, P-AKT, MMP-2, β-Catenin, Slug, P-β-Catenin, c-Myc, and Snail were not affected by ATG5 knockdown. Generally, downregulation of ATG5 could reverse the EMT process by specific pathways in cervical cancer cells and resulted in attenuation of migration and invasion.

## Discussion

Autophagy is an essential process induced under various conditions of cellular stress and tightly regulated to respond correctly to the ever-changing environment. It is now apparent that autophagy plays different roles in cancer progression. It plays a cancer inhibition role in the onset of tumorigenesis and cancer promotion in the cancer advancement phase (White, 2012). Autophagy inhibition may be an effective anti-cancer therapy in advanced cancer (Barbeau et al., 2020). Invasion and metastasis contribute to a high recurrence rate, which is the main cause of the poor prognosis of cancer patients (Malek et al., 2014). Despite advances in cancer management, some patients with cervical cancer undergoing early metastasis, especially lymph node metastasis, would ultimately lead to poor clinical outcomes (Nanthamongkolkul and Hanprasertpong, 2018; Xu et al., 2018). EMT, defined as a process by which cancer cells change from an epithelial phenotype to a mesenchymal phenotype, promotes invasion and metastasis by enhancing the capability of cancer cells to invade and migrate (Lamouille et al., 2014). Novel increasing evidence showed that the process of EMT could be promoted or inhibited by autophagy (Catalano et al., 2015; Dash et al., 2018; Daskalaki et al., 2018; Wang et al., 2019; Wu et al., 2019; Liu et al., 2020), and the core ARGs involved in the autophagy process plays important roles in EMT regulation (Peng et al., 2013; Liu et al., 2020).

In this study, we analyzed the prognosis of ARG signature in cervical cancer patients. By univariate and multivariate analysis, ATG5, ATG4D, and ATG4A were distinguished as independent prognostic predictors from the TCGA database. It was vital that ATG5 was the only important harmful marker influencing the prognosis of cervical cancer patients. ATG5 is one of the essential regulators of autophagy. Regardless of stage, grade, and pathological characteristics, cervical cancer patients with high expression of ATG5 had shorter survival. Due to the limited sample size (29 cases), there was no statistical significance in the adenocarcinoma group. However, the KM curve of the high-ATG5 group was significantly lower than that of the low-ATG5 group. Our results suggested that ATG5, as one of ARG signatures, was an essential contributor to the poor prognosis of cervical cancer. The higher expression of ATG5 was also associated with poor clinical outcomes of other cancers (Yang et al., 2017; Cheng et al., 2019). Conversely, positive expression of ATG5 predicts a favorable prognosis in patients with breast cancer and osteosarcoma (Wang et al., 2015; Zhao et al., 2018).

Besides the expression profile of ATG5, the hereditary genetic polymorphisms of ATG5 were also recognized as prognostic predictors of early-stage ESCC patients (Yang et al., 2017). Therefore, we investigate the genomic alterations and methylation of ARGs in CESC. Eight methylation sites of ATG5, namely, four sites with good prognosis and four sites with poor prognosis, were explored to be significantly associated with OS of cervical cancer patients. Four sites with beneficial OS (cg00333154, cg15336269, cg02711268, and cg21148531 located at chr6:106774171:106809238 length ≈35 kbp 10 based on illuminaMethyl450_hg19_GPL16304_TCGAlegacy annotation data) and four sites with harmful OS (cg24211478, cg10566763, cg26178529, and cg10850197 located at chr6:106773226:106773720 length 494 bp) were located in different positions of transcription regions of ATG5. The relation between DNA methylation and gene expression is importantly subjected to the genomic location of DNA methylation, which also affects the clinicopathological characteristics of cancers (van Vlodrop et al., 2011). Traditionally, cancer research predominantly focused on the prognostic significance and effects of promoter CpG island hypermethylation located in tumor suppressor genes (Jacinto et al., 2007; Amara et al., 2008; Singh et al., 2020). It is well known that DNA methylation of promoters can silence genes while growing evidence suggests that methylation may also be associated with gene activation (Smith et al., 2020). Moreover, methylation within a gene-body or transcribed region does not block the expression of the affected gene (Salem et al., 2000; Zilberman et al., 2007; Yang et al., 2014). These might explain the different prognostic significance among the eight methylation sites of ATG5. In addition, our analysis about genomic alterations and methylation of ATG5 showed the concomitant occurrence of ATG5 mutation and ULK2 mutation and indicated that methylation sites of ATG5 could also serve as prognosis predictors in cervical cancer patients. As reported by others, the inhibition of autophagy by promoter methylation of ULK2, which leads to downregulation of transcript levels, was essential for cancer growth under the genetic background of ATG5 (Shukla et al., 2014). Moreover, ATG5 was identified as one of the immune-related genes in ESCC (Li et al., 2017). Likewise, our study found that ATG5 was significantly correlated with immune-related genes involved in the immune effector process and regulation of T-cell-mediated immunity. Research has found that ATG5 silencing could abolish the EMT promotion by miR-210-5p in osteosarcoma (Liu et al., 2020). Also, in hepatocellular carcinoma, ATG5 downregulation drastically dampened TGF-β2-induced EMT (Dash et al., 2018). By contrast, knockdown of ATG5 increased migration and invasion in glioblastoma cells and RAS-mutated cancer cells by promoting EMT (Catalano et al., 2015; Wang et al., 2019). However, the effect of ATG5 on EMT and the prognosis in cervical cancer is unknown to date. We analyzed the relationship between ATG5 and EMT-related gene signature in cervical cancer. In addition, cervical cancer cell lines were used to investigate the effect of ATG5 on migration and invasion.

ATG5 knockdown could inhibit migration and invasion of cervical cancer cells by reversing EMT. N-cadherin, Vimentin, P-ERK, P-NFκBp65, P-mTOR, MMP-9, Fibronectin, and Twist might be the possible mechanisms involved in ATG5-dependent EMT regulation. However, function acquisition and loss of these genes were needed to be carried out in our further epidemical investigation. MMP-9, as a Matrix metalloproteinase, can destroy the integrity of the basement membrane and extracellular matrix and promote the detachment of tumor cells from the endothelium, which will lead to metastatic process (Voura et al., 2013; Rempe et al., 2016). Fibronectin is one of the ECM components and serves as a mesenchymal marker (Paolillo and Schinelli, 2019). Elevated protein expression of N-cadherin and Vimentin promotes mesenchymal phenotypic transition (Paolillo and Schinelli, 2019). ERK-, NFκBp65-, and mTOR-signaling pathways were the important mechanisms implicated in the regulation of the EMT process (Singh et al., 2018).

## Conclusion

**FIGURE 11:**
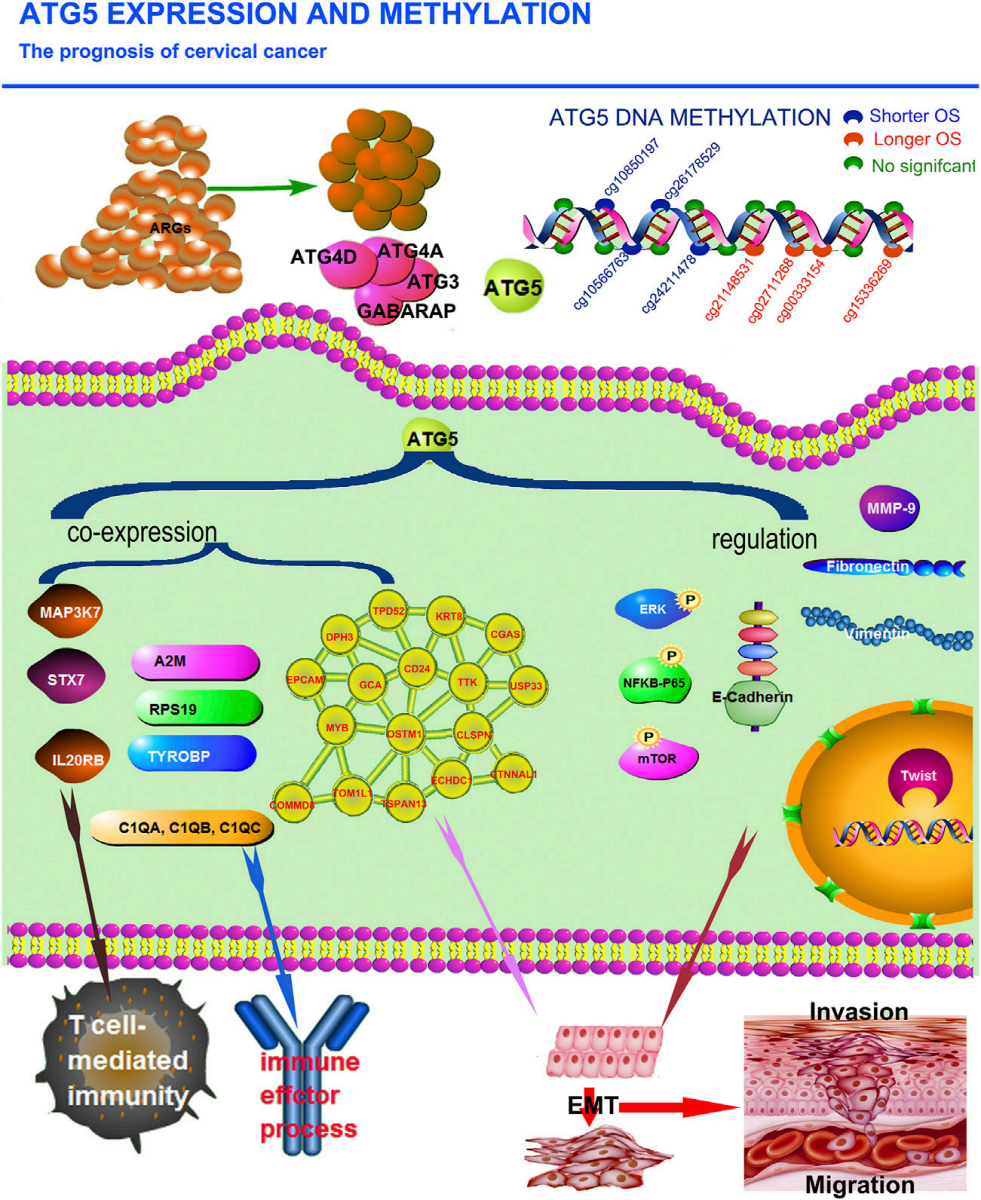
The illumination of the effect of ATG5 expression and methylation in cervical cancer. ATG5 among these 32 ARGs was the most significant harmful factor affecting the prognosis of CESC patients. ATG5 co-expressed genes involved in the regulation of the immune effector process were A2M, C1QA, C1QB, C1QC, RPS19, MAP3K7, TYROBP, STX7, and IL20RB. And, MAP3K7, STX7, and IL20RB were in-volved in the regulation of T cell-mediated immunity. Moreover, ATG5 might promote the migration and invasion of CESC by the regulation of N-cadherin, Vimentin, P-ERK, P-NFκBp65, P-mTOR, MMP-9, and Twist. ATG5 was also significantly correlated with EMT signature related genes including DPH3, CD24, KRT8, CTNNAL1, EPCAM, TSPAN13, MYB, TPD52, ECHDC1, CLSPN, USP33, TTK, TOM1L1, GCA, COMMD8, CGAS, and OSTM1. ATG5 methyltaion status also influence CESC OS. Among these 19 methylation sites in ATG5, four sites significantly increased while other four sites significantly decreased OS of cervical cancer patients.

In summary, ATG5 involved in the EMT process and immune regulation in cervical cancer could affect the survival of cervical cancer patients by expression and methylation level, proposing that ATG5 may be a potentially powerful therapeutic target for cervical cancer (Figure 11).

## Data Availability

The original contributions presented in the study are included in the article/supplementary material, further inquiries can be directed to the corresponding author.
