# Corrigendum: Increased ATG5 Expression Predicts Poor Prognosis and Promotes EMT in Cervical Carcinoma

**DOI:** 10.3389/fcell.2021.839706

**Published:** 2022-01-11

**Authors:** Suna Zhou, Xuequan Wang, Jiapei Ding, Haihua Yang, Youyou Xie

**Affiliations:** ^1^ Laboratory of Cellular and Molecular Radiation Oncology, The Affiliated Taizhou Hospital, Wenzhou Medical University, Taizhou, China; ^2^ Department of Radiation Oncology, The Affiliated Taizhou Hospital, Wenzhou Medical University, Taizhou, China

**Keywords:** autophagy-related genes, ATG5, autophagy, EMT, cervical cancer, prognosis

In the published article, we neglected to include the funder “This study was supported by Program of Taizhou Science and Technology Grant (20ywa04)”.

The authors apologize for this error and state that this does not change the scientific conclusions of the article in any way. The original article has been updated.

