# Supplementary figures and images for: Increased ATG5 Expression Predicts Poor Prognosis and Promotes EMT in Cervical Carcinoma
Source: Front Cell Dev Biol. 2021 Nov 25;9:757184. doi: 10.3389/fcell.2021.757184 (PMC8655861; doi:10.3389/fcell.2021.757184)

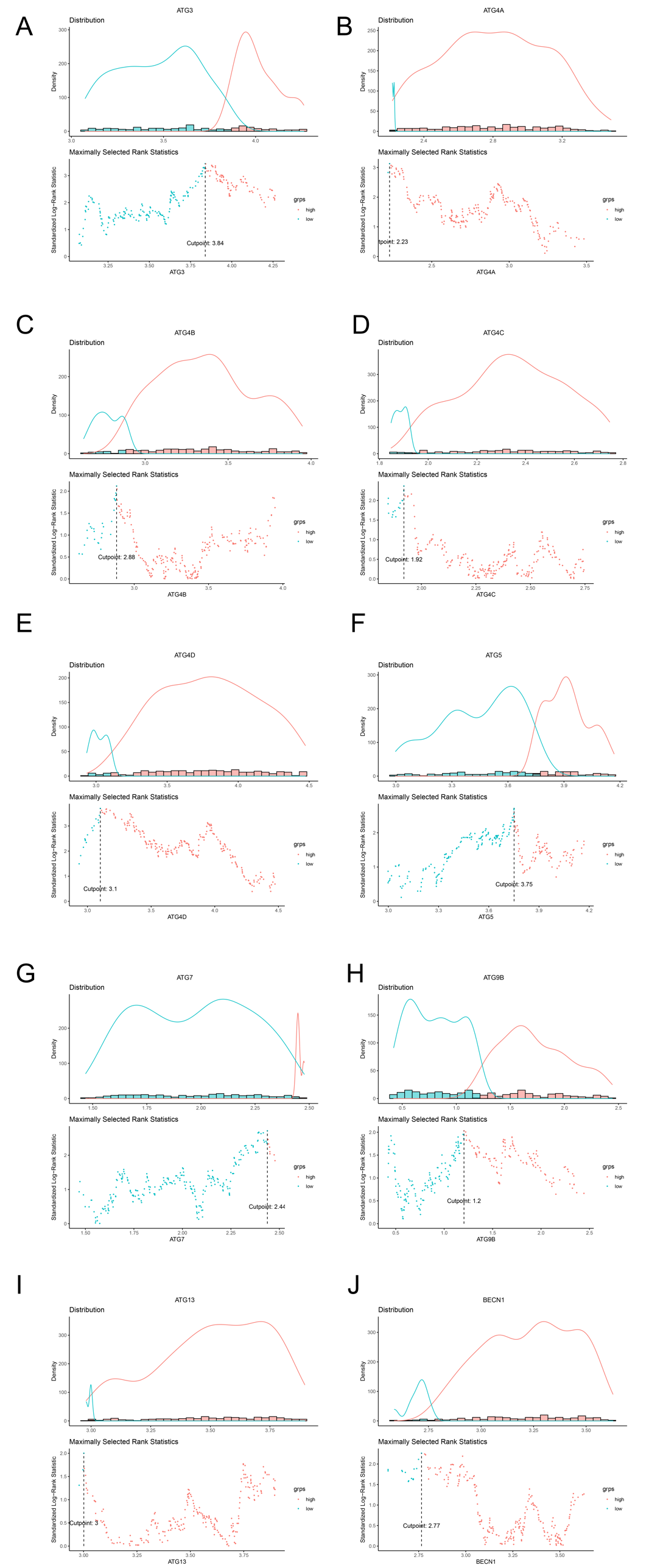

Supplement: Supplementary file 1 [file Image1.TIF]
